# MicroRNA miR-98 inhibits tumor angiogenesis and invasion by targeting activin receptor-like kinase-4 and matrix metalloproteinase-11

**DOI:** 10.18632/oncotarget.717

**Published:** 2012-11-06

**Authors:** Vinayakumar Siragam, Zina Jeyapalan Rutnam, Weining Yang, Ling Fang, Linlin Luo, Xiangling Yang, Minhui Li, Zhaoqun Deng, Jun Qian, Chun Peng, Burton B. Yang

**Affiliations:** ^1^ Sunnybrook Research Institute, Sunnybrook Health Sciences Centre; ^2^ Departments of Biology, York University; ^3^ The Affiliated People's Hospital of Jiangsu University, Zhenjiang City, China; ^4^ Department of Laboratory Medicine and Pathobiology, University of Toronto

**Keywords:** microRNA, miR-98, angiogenesis, tumorigenesis, invasion

## Abstract

Angiogenesis and invasion are essential processes for solid tumor growth and dissemination. The tumor development process can be dependent on the activation of a series of signaling pathways, including growth factor-activated pathways. MicroRNAs have been shown to be critical for tumorigenesis, but their roles in cancer angiogenesis, invasion and other signaling pathways important for tumor development are still unclear in the context of tumor biology. We investigated the role of microRNA miR-98 in regulating tumor growth, invasion, and angiogenesis using a highly aggressive breast cancer model in vitro and in vitro. We found that the expression of miR-98 inhibited breast cancer cell proliferation, survival, tumor growth, invasion, and angiogenesis. Conversely, inhibition of endogenous miR-98 promoted cell proliferation, survival, tumor growth, invasion, and angiogenesis. It appeared that miR-98 inhibited angiogenesis by modulating endothelial cell activities including cell spreading, cell invasion and tubule formation. Interestingly, miR-98 reduced the expression of ALK4 and MMP11, both of which were potential targets of miR-98. Transfection of an anti-miR-98 construct increased the expression of both targets. We confirmed that mir-98 targeted the 3'-untranslated regions of ALK4 and MMP11. Finally, ALK4- and MMP11-specific siRNAs inhibited breast cancer cell proliferation, survival, and angiogenesis. Rescue experiments with ALK4 and MMP11 constructs reversed the anti-proliferative, anti-invasive and anti-angiogenic effects of miR-98. Our findings define a regulatory role of miR-98 in tumor angiogenesis and invasion through repressed ALK4 and MMP11 expression.

## INTRODUCTION

The transforming growth factor-β (TGF-β) superfamily is a group of multifunctional proteins involved in diverse biological processes, including cell proliferation, differentiation, as well as inflammation [1]. Members of TGF-β superfamily are known to have diverse effects on tumor development, metastasis and angiogenesis [2]. Activin receptor-like kinase, ALK4 (also known as ActRIB) is a type 1 receptor of the serine/threonine kinase receptor family that mediates signalling induced by several members of the TGF-β superfamily, such as activins, Nodal, growth and differentiation factor (GDF)-1 and GDF-11 [3]. Upon activation, this type I receptor phosphorylates Smad proteins (SMAD2 and SMAD3), which then forms a complex with SMAD4 and enters the nucleus to regulate target gene expression [2, 4].

MicroRNAs (miRNAs) have emerged as a major class of gene regulators linked to a wide variety of biological functions. As a new class of regulatory molecules, miRNAs have diverse functions in regulating cell activities associated with cell proliferation [5-7], cell cycle progression [8, 9], differentiation [10, 11], invasion [12, 13], tumor growth [14-16], and metastasis [17-19]. MicroRNAs post-transcriptionally regulate gene expression through imperfect base pairing with sequences in the 3'-untranslated region (3'UTR) of the target mRNA, inducing mRNA degradation or translational repression [20, 21]. More than 1000 miRNAs have been identified in mammals; however, the functions of most of these miRNAs have not yet been elucidated.

MicroRNAs can also play a role in angiogenesis [22-26]. Work from our group indicates that miR-378 promotes angiogenesis by repressing Sufu and Fus1 expression [23] and that miR-93 is involved in angiogenesis by targeting integrin-β8 [24]. MiR-24 has also been shown to regulate erythroid differentiation by targeting the expression of ALK4, an activin type 1 receptor, demonstrating the potential role of miR-24 in activin signalling [27]. Furthermore, Hebert and co-workers [28] have reported that HMGA2 (High Mobility Group A2) expression in head and neck squamous cell carcinoma (HNSCC) cells are regulated by the expression of miR-98. In response to the microbial challenge, miR-98 has also been implicated to regulate CIS (Cytokine-inducible Src homology 2-containing protein) protein expression in human cholangiocytes [29]. Up to this point, the functional roles of miR-98 in angiogenesis and invasion have not previously been investigated. In this study, we report that miR-98 interferes with tumor invasion and angiogenesis by repressing ALK4 and MMP11 expression.

## RESULTS

### Proliferation and survival of breast cancer cells is affected by miR-98

**Figure 1:**
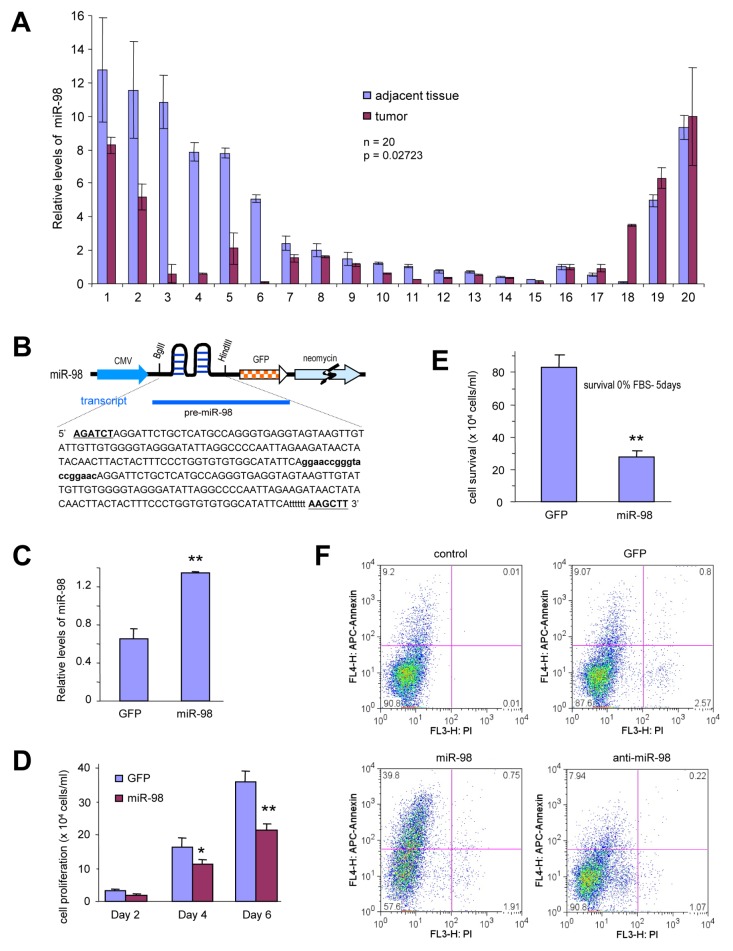
miR-98 construct generation, expression, and its effect on cell functions (A) RNAs were isolated from paraffin blocks of human breast carcinoma specimens and the normal breast tissues followed by real-time PCR analysis of miR-98 levels. The breast carcinoma tissues expressed higher levels of miR-98 than the normal tissues. (B) Structure of construct containing pre-miR-98, GFP, and neomycin. The bolded and capitalized letters indicate two restriction sites (BglII and HindIII). The bolded lower case sequence indicates an artifact sequence inserted between two pre-miRNAs. Six ‘t’ were added to stop the transcription. (C) RNAs were isolated from breast cancer cells 4T1 cells stably transfected with miR-98 or control vector GFP and subjected to real-time PCR to measure the expression of mature miR-98. (D) Breast cancer cells 4T1-cells stably transfected with miR-98 or a control vector GFP were seeded on tissue culture plates in medium containing 2.5% FBS and subjected to proliferation assays. The cell number was counted on day 2, 5 and 7. Data are expressed as mean ± SEM (n=4). *, *P* < 0.05. (E) The cells were maintained in serum-free conditions (0% FBS) for 5 days. Cell survival was assayed by counting the viable cells. Error bars indicate SEM (n=4) **P < 0.01. (F) The cells were also maintained in serum-free conditions for 4 days for apoptotic analysis. Transfection with miR-98 promoted apoptosis (39.8%) compared with the control (9.2%).

To determine the role of miR-98 in tumorigenesis, we analyzed the expression of miR-98 in human breast tumor tissues and normal breast tissues. RNAs were isolated from frozen metastatic tumor tissues and their adjacent benign tissues. Real-time PCR analysis indicated that in a total of ten paired specimens, seven pairs showed higher levels of miR-98 in the benign tissues than in the metastatic tumors. Two pairs showed higher levels of miR-98 in the tumor tissues and one pair did not produce any detectable difference (Fig 1A). These experiments suggested that miR-98 may play a role in breast cancer development.

To further study the role of miR-98, we generated a construct expressing pre-miR-98 (Fig 1B). The construct was stably expressed in 4T1, a breast cancer cell line. Expression of miR-98 was confirmed by real-time PCR. The relative levels of miR-98 were significantly higher in the miR-98-transfected cell line than in the control cell lines (Fig 1C).

4T1 cells stably transfected with miR-98 or a control vector were subject to proliferation assays in 2.5% serum-containing media. The proliferation rate was examined on days 2, 4 and 6. The cells expressing miR-98 showed reduced proliferation compared with the cells expressing the control vector (Fig 1D). The cells were also cultured in serum-free media for 5 days. Under these conditions, the survival of 4T1 cells expressing miR-98 was significantly reduced as compared with the control (Fig 1E). To further analyze the effect of miR-98 on cell survival, apoptotic analysis was performed. By fluorescence-activated cell sorting (FACS), we detected that transfection with miR-98 greatly promoted apoptosis (Fig 1F). Using an anti-mir-98 expression construct, the effects of an antisense sequence against miR-98 on cell apoptosis were also tested. Transfection of the anti-miR-98 only slightly decreased apoptosis as compared with the control. Cell proliferation assays were also conducted in the human breast cancer cell lines MDA-MB-231 (Fig S1A) and MDA-MB-468 (Fig S1B). Similar results were obtained, confirming that miR-98 inhibited, while anti-miR-98 enhanced proliferation of these cells. Through cell survival experiments, it was also confirmed that miR-98 inhibited, while anti-miR-98 enhanced survival of MDA-MB-231 (Fig S1C) and MDA-MB-468 (Fig S1D) cells.

**Figure 2:**
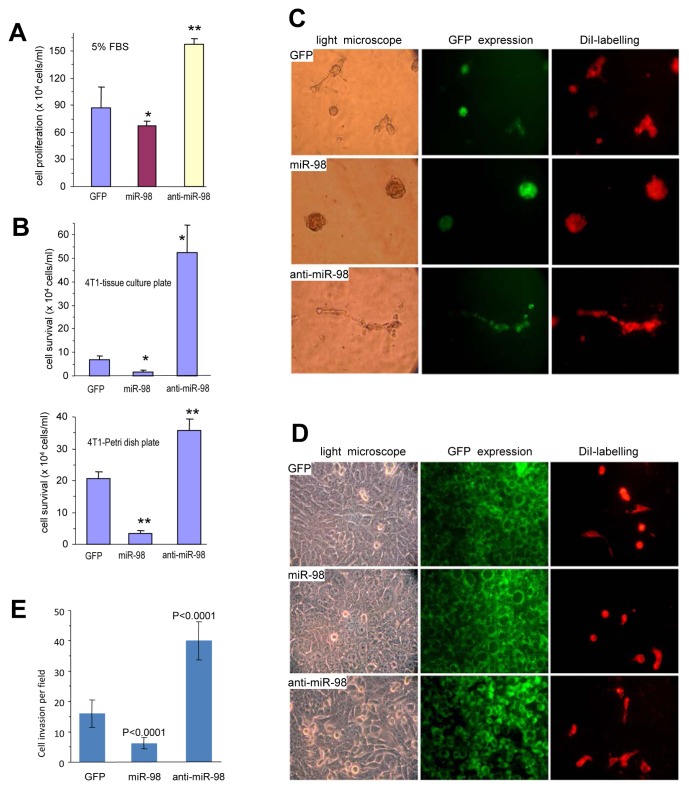
Effects of miR-98 on cell activities (A) 4T1 cells stably transfected with miR-98, anti-miR-98, or control GFP were seeded on tissue cultures plates containing 5% FBS for six 6 days for proliferation assays. *P < 0.05, **P< 0.01. Error bars, SEM (n=4). (B) The cells were seeded on tissue cultures plates (Upper) or Petri dishes (Lower) in serum-free conditions. Cell survival was monitored by counting the viable cells. **P< 0.01. Error bars indicate SEM (n=4). (C) The cells were mixed with Ypen cells and inoculated in Matrigel, followed by examination of tube formation. The Ypen cells formed larger complexes and longer tubes when mixed with the anti-miR-98-expressing cells compared with the GFP- and miR-98-transfected cells. (D) The cells, which had been labeled with green fluorescent dye DiO, were seeded on tissue culture plates. After overnight culture, endothelial Ypen cells, labeled with red fluorescent dye DiI, were inoculated on the top of the stably transfected cells. After 24 hours of co-culture, the Ypen cells cells were able to spread on the anti-miR-98 cells, but not on the GFP and miR-98 cells. (E) The cells were inoculated onto Matrigel in trans-well inserts. Three days after inoculation, the cells were stained with Coomassie Blue to examine cell invasion. The cells expressing anti-miR-98 exhibited stronger invasive activity than the others.

To further demonstrate the anti-proliferative effect of miR-98, the effects of antisense-miR-98 on cell proliferation and survival were tested. 4T1 cells transfected with miR-98, anti-miR-98 or control vector were seeded into tissue culture dishes containing 1%, 2.5%, or 5% serum. Expression of anti-miR-98 enhanced cell proliferation significantly compared with both the control cells and cells over-expressing miR-98 (Fig 2A). Expression of anti-miR-98 also enhanced cell survival under serum-free conditions compared with the control cells and miR-98 over-expressing cells (Fig 2B, Fig S2A).

### MiR-98 affects endothelial cell activity and invasion

It has been reported that miR-98 expression affects tumor growth [30]. We tested the effect of miR-98 on endothelial cell activity. The miR-98, anti-miR-98, or GFP-transfected cells were mixed with YPEN rat prostate endothelial cells and cultured in Matrigel to examine tubule formation. In the presence of the anti-miR-98-transfected cells, YPEN cells formed larger complexes and longer tube-like structures compared with both the GFP and miR-98-transfected cells (Fig 2C, Fig S2B). Similar results were obtained when MT-1 human breast cancer cells stably transfected with miR-98, anti-miR-98, or GFP were mixed with YPEN cells and cultured in Matrigel (Fig S2C).

4T1 cells stably transfected with GFP, miR-98, or anti-miR-98, were labeled with the green fluorescent dye DiO and seeded onto tissue culture plates overnight. Following inoculation of YPEN cells labeled with the red fluorescent dye DiI, the mixed cultures were examined by light and fluorescent microscopy. After an additional overnight culture, the endothelial YPEN cells were not able to spread over the miR-98, vector, or GFP cultures, but could spread more rapidly over the anti-miR-98 transfected cells (Fig 2D).

To further examine the role of mir-98 on cell invasion, the miR-98, anti-miR-98, or GFP-transfected 4T1 cells were inoculated on Matrigel in trans-well inserts and the cells that invaded through the inserts were examined. It was found that expression of miR-98 inhibited cell invasion while the expression of anti-miR-98 promoted cell invasion as compared with the cells transfected with GFP (Fig 2E, Fig S2D). These results indicated that mir-98 expression could inhibit endothelial cell activities and invasion. Cell morphology following anti-miR-98 transfection changed relative to the cells transfected with both the control vector and miR-98 in Petri dishes. Photographs of the cells expressing control, miR-98 and anti-miR-98 are shown in the Fig S2E.

### MiR-98 inhibits tumorigenesis and angiogenesis

**Figure 3:**
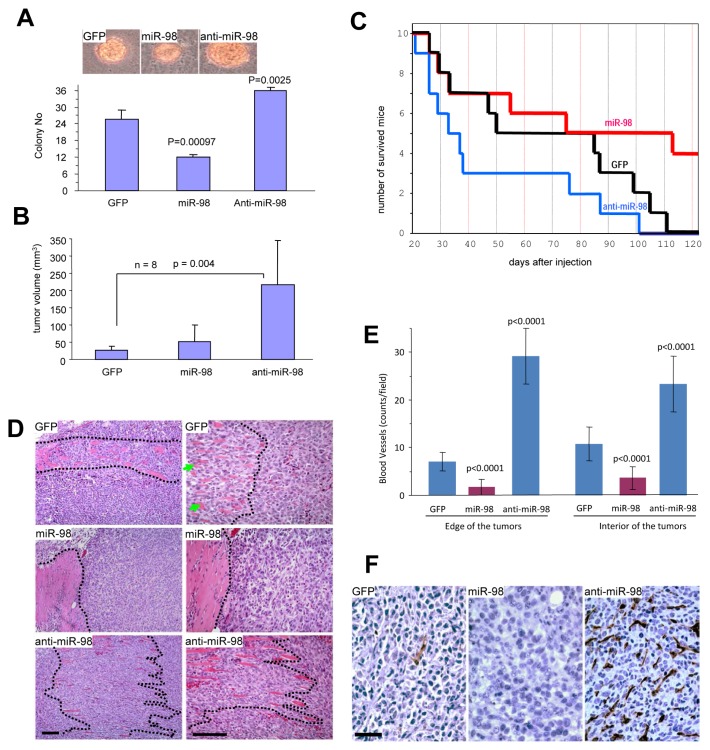
Anti-miR-98 promotes tumor formation, invasion, and angiogenesis (A) 4T1 cells (10^3^) were mixed with 0.3% low melting agarose containing 10% FBS and plated on 0.66% agarose-coated 6-well plates. Four weeks after cell inoculation, colonies were examined and photographed. Large colonies and smaller colonies within the cell complexes were counted. Typical large colonies from each group are shown (Lower Panel). (B) The cells were injected subcutaneously into Balb/c regular mice. Tumor growth was monitored. Expression of anti-miR-98 promoted tumor growth. (C) Animal viability was analyzed. (D) Tumors formed by the cells were subjected to H&E staining. Invasion of the tumor cells with stromal muscles (marked by dotted lines) occurred extensively in the anti-miR-98 cells than GFP cells. The miR-98 cells showed little invasive activity. scale bars, 100 μm. (E) The tumor sections were subjected to immunohistochemistry probed with anti-CD34 antibody to detect blood vessels. The number of blood vessels was counted in 10 randomly selected imaging fields and statistical analysis. Expression of miR-98 inhibited blood formation while expression of anti-miR-98 promoted this process. n=10. (F) At higher magnification, large number of vacuoles, sign of unhealthy and dead cells, could be detected in the miR-98 tumor, but not in the other two groups. scale bar, 40 μm.

In order to test the role of miR-98 in tumorigenesis, we performed colony-formation assays. 4T1 cells stably transfected with miR-98, anti-miR-98, or the control vector were plated in low melting agarose with 2% serum. These conditions allowed cells to expand and form 3-dimensional colonies. The cells expressing anti-miR-98 formed larger colonies and a greater number of colonies per plate compared with control cells or cells over-expressing miR-98 (Fig 3A). To further confirm the effects of miR-98 on tumorigenesis, cell lines transfected with miR-98, anti-miR-98, or the control vector were injected subcutaneously into Balb/c mice. Tumor formation was monitored and tumor sizes were measured regularly. 4T1 cells expressing anti-miR-98 developed tumors at a greater rate than cells expressing the mir-98 or control vector (Fig 3B, Fig S3) and had to be sacrificed earlier due to open tumor wounds (Fig 3C). Due to the rapid growth rate of 4T1 cells, tumor formation frequently led to open wounding in the mice. As mandated by the Sunnybrook Animal Care Committee, these mice were sacrificed. Tumor samples were then sectioned for histological analysis. Along the tumor boundaries, local invasion of the tumor into the surrounding stromal smooth muscle was detected, especially in anti-miR-98 tumors as compared to control tumors. The expression of miR-98 inhibited local invasion compared with the control cells (Fig 3D).

The tumors were also tested for CD34 expression and cell death. The tumors formed by the miR-98-transfected cells displayed a significantly reduced amount of blood vessels while the tumors formed by the anti-miR-98-transfected cells contained larger and a significantly higher amount of blood vessels than those formed by the control vector (Fig 3E, Fig S4). The miR-98-derived tumor cells surrounding the blood vessels appeared apoptotic, containing vacuoles and either condensed or fragmented nuclei (Fig 3F). These results suggested that miR-98 could play a role in the inhibition of blood vessel formation and tumorigenesis. Consistent with this was the observation that the number of apoptotic cells was increased in the control and miR-98-derived tumors compared to anti-miR-98-derived tumors (Fig S5).

### MiR-98 represses ALK4 and MMP11 expression

**Figure 4:**
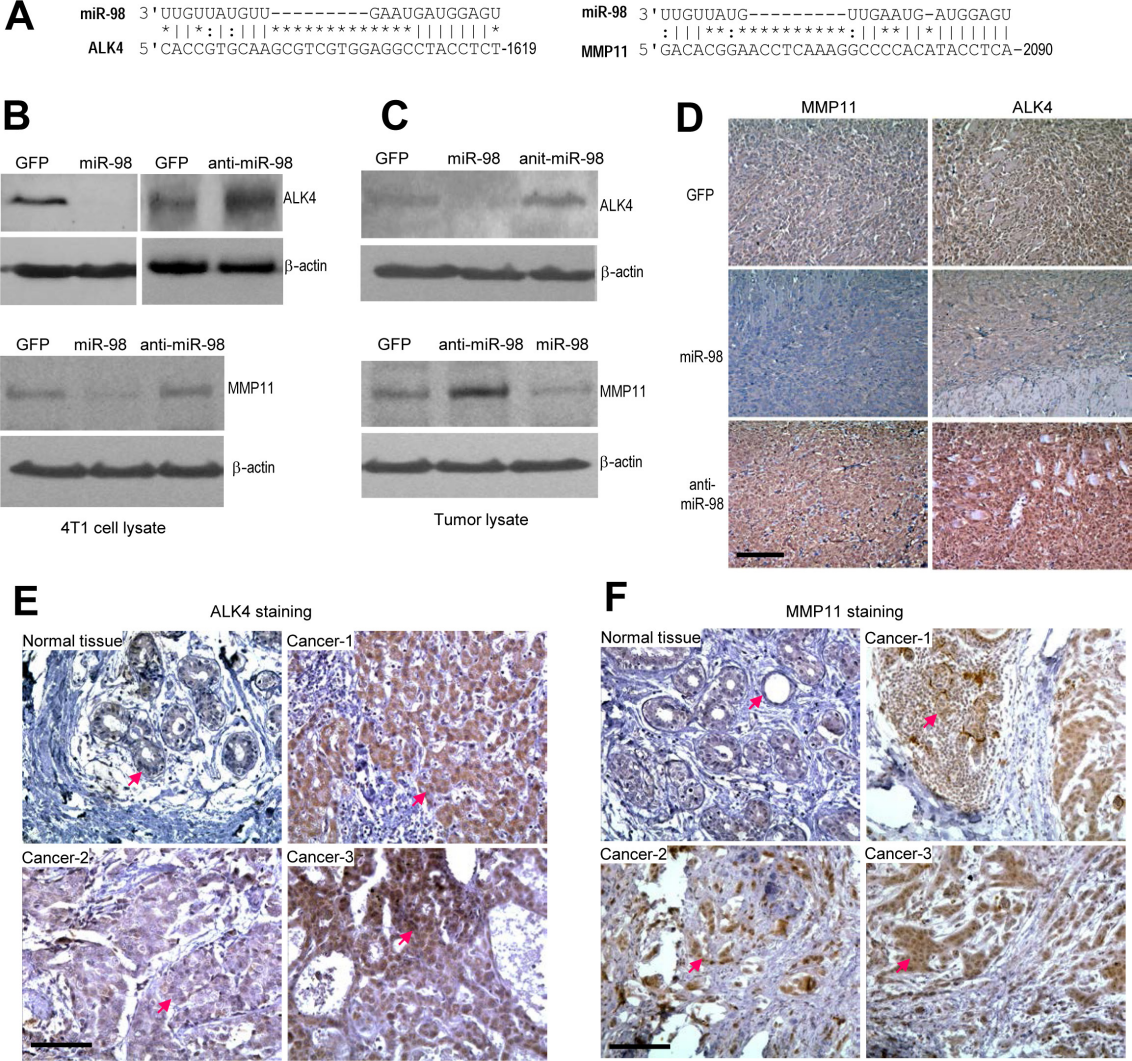
Targeting of ALK4 and MMP11 by miR-98 (A) Potential binding sites of miR-98 was found in the 3'UTRs of ALK4 and MMP11. (B-C) Protein lysates were prepared from the 4T1 cells stably transfected with mir-98, anti-miR-98, or control GFP (B), and tumors formed by these cells (C). The lysates were subject to Western blot analysis for expression of ALK4 and MMP11. The same membranes were probed for actin expression to confirm equal loading. Expression of miR-98 repressed ALK4 and MMP11 levels while expression of anti-miR-98 played opposite effects. (D) The tumor sections were immunostained with anti-ALK4 and MMP11 antibodies. The miR-98 tumors exhibited lower levels of ALK4 and MMP11 than GFP tumors, while the anti-miR-98 tumors showed higher levels of ALK4 and MMP11 than GFP tumors. scale bars, 100 μm. (E-F) Human breast carcinoma specimens and the adjacent normal tissues were probed with anti-ALK4 and MMP11 antibodies. The tumor areas expressed higher levels of ALK4 and MMP11 than the normal tissues.

To elucidate the mechanism of the observed miR-98 effects, we utilized computational approaches to identify putative binding sites for miR-98. MiR-98 binding sites were identified in various mRNAs, many of which were associated with tumor growth and invasion including Activin A receptor, type IB or ALK4, and Matrix metalloproteinase-11 or MMP11 (Fig 4A). We tested the expression of these proteins by Western blot analysis in cell lines stably transfected with miR-98, anti-miR-98, or GFP,as well as in the tumor lysates derived from these cell lines. We found that the expression of ALK4 and MMP11 was repressed in mir-98 expressing cells (Fig 4B) and in miR-98 derived tumors (Fig 4C) as compared with the cells transfected with GFP and the GFP tumors. Conversely, the expression of ALK4 and MMP11 was up-regulated in the cells expressing anti-miR-98 and in the anti-miR-98 tumors as compared with the cells transfected with GFP and the GFP tumors. By immunohistochemistry, we then examined the localization of these proteins and found that the repression by miR-98 and up-regulation by anti-miR-98 was evenly distributed across the tumor sections (Fig 4D). To examine whether there was correlation between the expression of ALK4 and MMP11, we examined expression of these proteins in human breast carcinoma specimens. We found that expression of ALK4 (Fig 4E) and MMP11 (Fig 4F) was much higher in human breast carcinoma specimens than in normal breast tissues.

**Figure 5:**
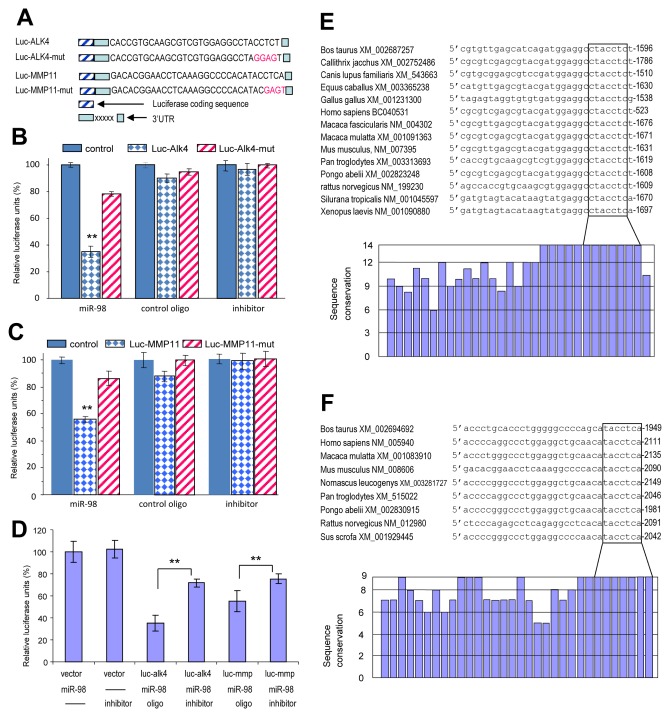
Luciferase assays confirmed that miR-98 could target ALK4 and MMP11 (A) Luciferase constructs containing the target sites were generated producing Luc-ALK4 and Luc-MMP11. Mutations were created in the potential target sequence (red) producing Luc-ALK4-mut and Luc-MMP11-mut. (B-C) 4T1 cells were co-transfected with the luciferase constructs of ALK4 (G) or MMP11 (H), the mutant constructs, or a control construct which contained a non-related fragment of versican G3 domain, with miR-98 mimic, miR-98 inhibitor (anti-miR-98), or an oligo with random sequence. Luciferase activities were determined. Luciferase activities decreased when the constructs were co-transfected with miR-98, which was reversed when the target sites were mutated (n=3, *P < 0.05, *P<0.01). (D) 4T1 cells were co-transfected with the luciferase constructs of ALK4 (luc-alk4) or MMP11 (luc-mmp) or the control construct, with miR-98 mimic, miR-98 inhibitor (inhibitor), or an oligo with random sequence. Luciferase activities decreased when the constructs were co-transfected with miR-98, which was significantly reversed when the miR-98 inhibitor was included (n=3, *P<0.01). (E) Upper, alignment of the miR-98 targeting ALK4 sequences across *Bos taurus* (Genbank access number following the name), *Callithrix jacchus, Canis lupus familiaris, Equus caballus, Gallus gallus, Homo sapiens, Macaca fascicularis, Macaca mulatta, Mus musculus, Pan troglodytes, Pongo abelii, Rattus norvegicus, Silurana tropicalis, Xenopus laevis*. The seed regions for miR-98-ALK4 interactions are in the box. Lower, conservation of the sequences is shown across all species. (F) Upper, alignment of the miR-98 targeting MMP11 sequences across *Bos taurus* (Genbank access number following the name), *Homo sapiens, Macaca mulatta, Mus musculus, Nomascus leucogenys, Pan troglodytes, Pongo abelii, Rattus norvegicus, Sus scrofa*. The seed regions for miR-98-MMP11 interactions are in the box. Lower, conservation of the sequences is shown across all species.

To confirm the targeting of ALK4 and MMP11 by miR-98, we cloned the 3'UTRs of ALK4 and MMP11 and inserted them into luciferase reporter constructs, producing constructs Luc-ALK4 and Luc-MMP11 (Fig 5A, Fig S6). The miR-98 target sites were also mutated to generate the mutant constructs, Luc-ALK4-mut and Luc-MMP11-mut. 4T1 cells were co-transfected with the luciferase construct Luc-ALK4, a control construct, or the mutant construct Luc-ALK4-mut, each combined with either a miR-98 mimic, miR-98 inhibitor (anti-miR-98), or a control oligo (with a random sequence). The experiments showed that miR-98 significantly decreased luciferase activity in the Luc-ALK4-transfected cells (Fig 5B). Mutation of the predicted miR-98 binding site abolished the inhibitory effect of miR-98 on Luc-ALK4-mut. We noted some reduction in luciferase activity when the cells were co-transfected with the luciferase construct Luc-ALK4 and the random sequence control, suggesting a functional effect of endogenous miR-98. Similar results of luciferase assays were obtained when the cells were co-transfected with the Luc-MMP11, Luc-MMP11-mut and the different oligos (Fig 5C). By luciferase assay, we also confirmed the knockdown efficiency of anti-miR-98. Addition of anti-miR-98 significantly reduced the inhibitory effect of miR-98 on luciferase activity as compared with the control oligo (Fig 5D). Examination of the target sequences indicated that the miR-98 target sites were highly conserved across different species. In sequences obtained from all species, the seed regions that were critical for miR-98 targeting were 100% homologous in ALK4 (Fig 5E) and MMP11 (Fig 5F).

### Confirmation of miR-98 functions by targeting ALK4 and MMP11

**Figure 6:**
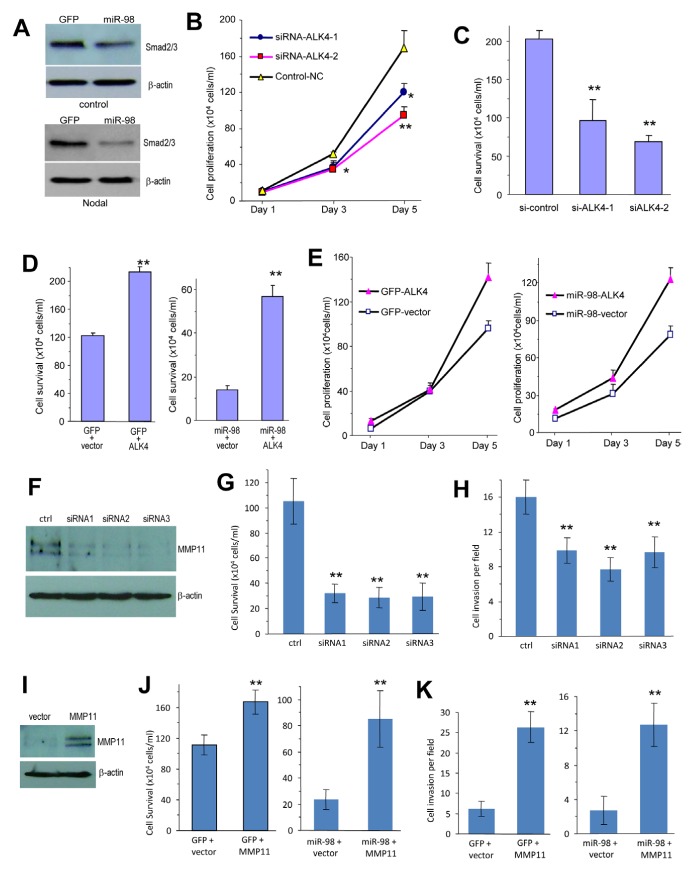
Confirmation of ALK4 and MMP11 functions (A) Cell lysates prepared from 4T1 cells transiently transfected with miR-98 or GFP and treated with or without Nodal were subjected to Western blot analysis probed with anti-phosphorylated Smad2/3 antibody. Staining for β-actin using the same membranes confirmed equal loading. (B) 4T1 cells transiently transfected with the siRNA or the control oligo were grown on 6-well tissue culture dishes in 5% serum containing medium. Cell proliferation rate was determined by counting the cells on day 1, 3 and 5. * *P* < 0.01, *P<0.01. Error bars, SD (n = 3). (C) 4T1 cells transiently transfected with the siRNAs or the control oligo were grown on 6-well tissue culture dishes in serum-free conditions. Cell survival was monitored with a light microscope. Surviving cells were harvested and counted. ** *P* < 0.01. Error bars, SD (n = 3). (D) 4T1 cells stably transfected with miR-98 or GFP were transiently transfected with ALK4 or a control vector followed by culturing in serum-free conditions for 5 days. Cell survival was assayed by counting the viable cells. *P < 0.05. Error bars indicate SEM (n=4). (E) 4T1 cells stably transfected with miR-98 or GFP control vector were transiently transfected with ALK4 or a control vector. The cells were grown on 12-well plates in 5% serum containing medium. The proliferation rate was examined on days 1, 3, and 5. ** *P* < 0.01. Error bars SEM (n=4). (F) Cell lysates prepared from 4T1 cells transiently transfected with siRNAs targeting MMP11 or a control oligo were analyzed on Western blot probed with anti-MMP11 antibody. (G) 4T1 cells transiently transfected with MMP11 siRNAs or the control oligo (ctrl) were grown on 6-well tissue culture dishes in serum-free conditions. Cell survival was monitored with a light microscope. Surviving cells were harvested and counted. ** *P* < 0.001. Error bars, SD (n = 10). (H) 4T1 cells transiently transfected with the siRNA or the control oligo. The cells were harvested and suspended in 100 μl serum-free DMEM medium, followed by inoculation onto Matrigel in trans-well inserts. Two days after inoculation, the cells were analyzed for cell invasion. The cells transfected with siRNAs exhibited weaker invasive activity than the cells transfected with a control oligo ** *P* < 0.001. Error bars, SD (n = 10). (I) Cell lysate prepared from MMP11- or a control vector-transfected 4T1 cells that had been stably transfected with miR-98 or the control GFP were subjected to Western blot analysis probed with anti-MMP11 antibody. Staining for β-actin from the same membrane confirmed equal loading. (J) 4T1 cells stably transfected with miR-98 or GFP were transiently transfected with MMP11 or a control vector followed by culturing in serum-free conditions for 5 days. Cell survival was assayed by counting the viable cells. *P<0.001. Error bars indicate SEM (n=12). (K) 4T1 cells stably transfected with miR-98 or GFP were transiently transfected with MMP11 or a control vector. The cells, harvested and suspended in 100 μl serum-free DMEM medium, were loaded into the Matrigel coated insert and incubated at 37°C for 48 hours fro invasion assay. Expression of MMP11 promoted cell invasion. **, *p<* 0.001. Error bars indicate SD (*n=*10).

To confirm that miR-98 effects were mediated through ALK4, we transfected 4T1 cells transiently with miR-98 and GFP and analyzed Smad2/3 levels. Down regulation of Smad2/3 was detected in the miR-98-transfected cells but not in the GFP-transfected cells (Fig 6A). In addition, the cells were transiently transfected with siRNAs against ALK4 causing down-regulation of the ALK4 protein (Fig S7A). It was found that ALK4-targeting siRNA reduced cell adhesion (Fig S7B), cell proliferation (Fig 6B), cell survival (Fig 6C, Fig S7C), and endothelial tube formation (Fig S7D). These results indicated that ALK4 played important roles in mediating these activities in 4T1 cells. They also suggested that miR-98 could reduce cell adhesion, cell proliferation, cell survival and endothelial cell activities through the down regulation of ALK4.

To further examine the role of ALK4 in mediating the function of miR-98, we conducted rescue experiments. 4T1 cells stably transfected with miR-98 were transiently transfected with an expression construct of ALK4 or a control vector (pcDNA4). Cell survival and proliferation assays indicated that the re-introduction of ALK4 into the miR-98-transfected cells enhanced cell survival (Fig 6D) and proliferation (Fig 6E). Western blot confirmed the over-expression of ALK4 (Fig S7E). Over-expression of ALK-4 also increased endothelial tube formation of 4T1 cells compared to the control cells (Fig S7F). These results suggested that the re-expression of ALK4 in miR-98 cells enhanced cell survival, angiogenesis and stimulated the proliferation by ALK4 up-regulation.

To confirm that the miR-98 effects were mediated through MMP11, we transfected 4T1 cells transiently with siRNAs against MMP11. Transfection with siRNAs caused down-regulation of the MMP11 (Fig 6F). Furthermore, MMP11-targeting siRNA reduced cell survival (Fig 6G), cell invasion (Fig 6H, Fig S8A), and endothelial tube formation (Fig S8B). These results indicated that MMP11 played important roles in mediating these activities in 4T1 cells. They also suggest that miR-98 could reduce cell invasion, endothelial tube formation and cell survival through down-regulation of MMP11.

We also conducted rescue experiments by transfecting the miR-98 cells with MMP11 expression construct or a control vector. Western blot confirmed overexpression of MMP11 (Fig 6I). Cell survival and invasion assays indicated that the re-introduction of MMP11 into the miR-98-transfected cells enhanced cell survival (Fig 6J, Fig S8C) and invasion (Fig 6K, Fig S8D). Furthermore, endothelial cell activities were promoted in the MMP11 over-expressing cells (Fig S8E). These results suggested that miR-98 played an important role in regulating MMP11 effects in 4T1 cells.

## DISCUSSION

We utilized the breast cancer cell lines, 4T1, MT1, MDA-MB-231 and MDA-MB-468, to study the regulatory role of miR-98 in tumor growth, angiogenesis, and invasion. 4T1 is a highly aggressive tumor cell line, which forms tumors in normal mice, mimicking the ability of human breast cancer to form tumors in the presence of a functional immune system. We showed that miR-98 could inhibit cell survival, proliferation, tumorigenesis *and angiogenesis by d*own-regulating ALK4 and MMP11 expression. By subcutaneous injection of mice, we found that the miR-98 over-expressing cells had a reduced tumor growth rate as compared to both the control and anti-miR-98 cells. Furthermore, breast cancer cells expressing anti-miR-98 formed larger colonies compared to the miR-98-transfected cells *in vitro*. MiR-98-expressing tumors were nuclei-poor relative to both anti-miR-98 and control tumors, suggesting that cells expressing anti-miR-98 could proliferate faster than miR-98 transfected cells. Also, in the human metastatic breast cancer samples analyzed, higher amounts of miR-98 were found in the benign tissues as compared to the metastatic tumor tissues. Therefore, our study provided evidence that miR-98 possessed tumor suppressor activity in the breast cancer tissues and suggests that the repression of miR-98 may promote tumorigenesis.

The role of miR-98 on tumor angiogenesis is still unclear. There are some reports suggesting that miR-98 expression is associated with tumor cell growth [31-33]. Clinical studies also suggest that miR-98 expression affects head and neck cancer development [28] and is under-expressed in nasopharyngeal carcinoma [34]. However, miR-98 was also found to be up-regulated in primary breast cancer specimens confirmed by microarrays and real-time PCR [35]. These results suggest that miR-98 may function as a tumor suppressor. The results from clinical analyses suggest that miR-98 may function differentially in different types of cancers. Our studies reveal that mir-98 inhibits tumor angiogenesis by targeting activin receptors (ALK4). Our results indicated that ALK4 levels were inversely associated with miR-98 expression, thereby suggesting a potential role of this receptor in breast tumor progression. Our findings further indicated that miR-98 may suppress tumor growth by targeting ALK4. Moreover, ectopic expression of miR-98 could overcome resistance to apoptosis in our 4T1 model by repressing ALK4 expression. Cumulatively, our data suggest a novel mechanism by which miR-98 directly modulates ALK4 expression and consequently the invasion threshold of the cancer cells. Although we could not exclude the possibility that other miRNAs may have cooperated with miR-98 to inhibit tumor angiogenesis and invasion, our results suggested that miR-98 could serve as a novel potential maker for breast cancer therapy.

The expression of miR-98 appears to be pro-apoptotic. Analysis of tumor sections showed signs of cell death in the miR-98-expressing cells, while there were fewer signs of cell death in the control and anti-miR-98 tumors. The tumors expressing miR-98 could not expand as extensively as the anti-miR-98-expressing tumors, leading to extensive cell death in the miR-98-expressing tumors. These results were supported by *in vitro* experiments. When 4T1 cells transfected with miR-98 or anti-miR-98 were cultured in serum-free conditions, anti-miR-98-tranfected cells survived longer than miR-98-transfected cells, or control vector cells.

The role of miR-98 in inhibiting angiogenesis were supported by a number of *in vitro* experiments showing that miR-98 decreased endothelial cell activities including cell spreading and tubule formation. In the cellular spreading experiments, the endothelial cells YPEN were able to spread rapidly on top of the anti-miR-98-transfected cells but not on the GFP, or miR-98 transfected cells. These results strongly suggested that the surface of the anti-miR-98-transfected cells were different from those of the GFP or miR-98-transfected cells. MiR-98 may repress cell surface proteins expression, inhibiting both the spreading of endothelial cells and the close contact of endothelial cells with the miR-98-transfected cells. This function may also be related to the inhibition of invasion in miR-98 transfected cells.

We found that tubule formation was enhanced by anti-miR-98-transfected cells when co-cultured with Ypen cells. The increased tubule formation in Matrigel by Ypen cells co-cultured with the anti-miR-98-transfected cells was a strong indication of enhanced angiogenesis. When the cell number was low, extensive tubule formation did not occur. Larger complexes were seen in the presence of anti-miR-98 expressing cells. These results further confirmed that the miR-98-transfected cells were unable able to interact well with endothelial cells, inhibiting blood vessel formation. The miR-98 expressing cells could not facilitate endothelial cell activities associated with blood vessel formation and extension. These results strongly implicated miR-98 as a key modulator of angiogenesis.

To further understand the functions of miR-98, we utilized computational algorithms to identify potential targets of miR-98. We found that many of the identified mRNAswere associated with tumor growth and angiogenesis, but only three of them were repressed in miR-98-transfected cells. Two of them, ADAM15 and MMP11, were known to play important roles in tumor invasion [36, 37]. Inhibition of both MMPs and ADAMs has been shown to inhibit tumor progression [37, 38]. The proposed role of miR-98 in decreasing MMP11 and ADAM-15 expression can explain the tumorigenic properties of breast cancer cells. The observation that miR-98 expression inhibited tumor invasion could be explained by the down-regulation of MMP11. We confirmed that there was indeed a down-regulation of MMP11 in both miR-98-expressing cells and tumors. Furthermore, the MMP11 siRNA survival, invasion and angiogenesis experiments mimicked the effect of miR-98. Over-expression of MMP11 resulted in the increased survival, invasion and angiogenesis of the 4T1 cells.

The other potential target of miR-98, ALK4, is known to mediate the functions of several members of the TGF-b superfamily. Presently, there is limited evidence indicating that ALK4 plays a role in tumorigenesis. Nevertheless, among the ligands identified for ALK4, Nodal and activins may have a role in tumor development and angiogenesis, although their effects vary among different types and/or stages of cancer [39-41]. Nodal and activins have been detected in breast cancer cells and they can regulate cell proliferation and apoptosis [42, 43]. Although activins and Nodal can both signal through ALK4, binding of Nodal requires a co-receptor, Cripto-1, which also inhibits signalling of activins through ALK4 [44]. Since Cripto-1 is overexpressed in breast carcinoma and is known to promote breast cancer development, metastasis and angiogenesis [45], it is likely that Nodal is the ligand that activates ALK4 to enhance tumor angiogenesis.. To support our hypothesis, we conducted silencing experiment to knockdown endogenous ALK4 and performed a rescue experiment to transfect the miR-98 cells with an ALK4 expression construct. We showed that there was a decrease in cell proliferation and survival when the cells were transfected with ALK4 siRNAs. On the other hand, when the miR-98 expressing cells were transfected with an ALK4 expression construct, we detected an increase in cell proliferation and survival. These results confirmed that ALK4 was a target of miR-98 and mediated the functions of this miRNA. Our data strongly suggest that the tumor suppressing effects of miR-98 may be mediated by inhibitingthe ALK4 and MMP11 signaling pathways.

In summary, we have demonstrated that miR-98 functions as a tumor suppressor by inhibiting cell survival, cell proliferation, tumor growth, tumor invasion, and blood vessel expansion, primarily by targeting ALK4 and MMP11. We speculate that the inhibition of miR-98 may play a critical role in the development of cancer. Given the improved characterization of the miRNA family however, it is reasonable to expect that there are multiple miRNAs with both tumor-promoting and tumor-suppressing functions. The precise role of miRNA-mediated tumor suppression in a complex pathway of tumorigenesis in the context of different cancers awaits further investigation.

## MATERIALS AND METHODS

### Cell lines and transfection

Mouse breast cancer cell line 4T1 (American Tissue Culture Collection) was cultured in DMEM medium supplemented with 10% FBS, and antibiotics in a 5% CO_2_ atmosphere at 37°C. 3×10^5^ cells/well in 6-well culture plates were transfected with miR-98 or a control vector using Lipofectamine™ 2000 (Invitrogen, USA). Stably transfected cells were selected using G418 antibiotic (Calbiochem, San Diego, CA) at a final concentration of 400 μg/ml. On day 15 after transfection, cells were assayed for reporter gene activity.

### Generation of miR-98 and anti-miR-98 expression construct

To address the effect of miR-98 on cell functions, the miR-98 plasmid was synthesized by our lab. The pre-miRNA-98 was ligated into a mammalian expression vector, BluGFP, which contained a Bluescript backbone, a CMV promoter driving expression of green fluorescent protein GFP between the BglII and HindIII restrictions sites (Fig 1B).

To address the role of endogenous miR-98 in mediating cell functions, the anti-miR-98 plasmid was designed using a similar approach, an antisense sequence to miR-98 was inserted in the expression vector producing an anti-miR-98 construct. Briefly, the primer anti-miR-98 was designed to incorporate an anti-miR-98 sequence into the expression vector by PCR, followed by restriction digestion and ligation as above to produce the anti-miR-98 construct.

### Luc-ALK4 and Luc-MMP11 constructs

A luciferase reporter vector (pMir-Report, Ambion) was used to generate luciferase reporter constructs. Two primers for ALK4, musALK4-1-SacI (5'cccggggagctctaagctgttcctctgcctacac) and musALK4-R98-MluI (5'gggcccacgcgtgccgggcagaaacaagagg), were used to clone the fragment of ALK4 3'UTR by RT-PCR. The PCR product was digested with SacI and MluI, followed by insertion into a SacI- and MluI-opened pMir-Report vector. To generate a mutant construct containing a mutation in the miR-98 target sequence, a primer musALK4-R98-MluI-mut (5'gggcccacgcgtgccgggcagaaacaactcctagcc) was combined with the primer musALK4-1-SacI in a PCR. The PCR products were digested with SacI and MluI and inserted into a SacI- and MluI-opened pMir-Report vector.

Two primers MusMMP11-SacI (5' cccgagctctgacaacactttggatgcattcag) and MusMMp11-R98-MluI (5'cccacgcgtggcctggggcaaggctgtgag) were designed to clone the fragment of MMP11 3'UTR to generate a luciferase construct Luc-MMP11 in a similar way. To generate a mutant construct, the primer MusMMP11-R98-MluI-mut (5'cccacgcgtggcctggggcaaggctgactcgtatg) was combined with MusMMP11-SacI.

To silence endogenous ALK4, two siRNAs were used targeting the sequences at 5' AACTACACGTGTGAGACAGAT (si-ALK4-1) and 5' AATCAGAGGGTGGGGACCAAA (si-ALK4-2), which achieves a potent silencing of ALK4 [46]. To silence endogenous MMP11, three siRNAs, 5'gugcucaguacuggguauatt (si-MMP11-1), 5'ccugccugauguacugaautt (si-MMP11-2) and 5'gguaccuucugagauugautt (si-MMP11-3), were synthesized.

### RT- PCR and RNA analysis

Total RNAs were extracted from cell cultures with mirVana miRNA Isolation Kit (Ambion) according to the manufacturer's instructions. RT-PCRs were performed as previously described [47]. For mature miRNA analysis, total RNAs were extracted from ~1×10^6^ cells, followed by first strand cDNA synthesis using 1 μg RNA. PCRs were performed with QuantiMir-RT Kit. To perform these experiments, other kits were also needed including Qiagen, miScript Reverse Transcription Kit, cat#218060, miScript Primer Assay, cat#218411, and miScriptSYBR GreenPCR Kit, cat#218073. The primer specific for mature miR-98 was purchased from Qiagen. The primers used as real-time PCR controls are provided in Supplementary Information Table S1.

### Western blot analysis

Cell lysates were prepared from 4T1 cells expressing different constructs or extracted from frozen tumor tissues and subjected to SDS-PAGE electrophoresis on 10-12% separating gel with a 4% stacking gel section. The lysis buffer contained protease inhibitors (150 mM NaCl, 25 mM Tris-HCl, pH 8.0, 0.5 M EDTA, 20% Triton X-100, 8 M Urea, and 1x protease inhibitor cocktail). The proteins separated on SDS-PAGE were transferred onto a nitrocellulose membrane (Bio-Rad) in Tris-glycine buffer containing 20% methanol. The membrane was then blocked in TBST (10 mM Tris-Cl, pH 8.0, 150 mM NaCl, 0.05% Tween-20) containing 5% skim milk powder (TBSTM) for 1 hour at room temperature with gentle shaking. The membrane was then incubated at 4 °C overnight with primary antibody. Next day, the membranes were washed with TBST (4×15 min) and incubated at room temperature with secondary anti-mouse or anti-rabbit antibody conjugated to horseradish peroxidase in TBSTM. After washing as above, the bound antibodies were visualized with the Chemiluminescent HRP Antibody Detection Kit (HyGLO, Denville Scientific Inc.).

### Luciferase activity assays

Luciferase activity assays were performed using the Promega luciferase Assay System as described previously [48]. In brief, 4T1 cells were cultured in 24-well tissue culture plates in DMEM containing 10% FBS. The cultures were co-transfected with the luciferase reporter constructs, corresponding miRNA mimics, and Renilla luciferase construct by Lipofectamine 2000. The cells were then collected and lysed with luciferase-specific lysis buffer from a Luciferase Assay Kit (Promega, Nepean, ON, Canada). The mixtures of cell lysates were centrifuged at 5000 rpm for 5 min. The supernatant was collected and used to measure firefly luciferase activities using a luminometer. For the internal control, 100 μl of Stop & Go reagent was added to the samples and Renilla luciferase activities were then measured in the same tube. Luciferase activities between different treatments were compared after normalization with Renilla luciferase activities.

### Cell survival assay

Cells were seeded on 35-mm Petri dishes or 6 well tissue culture dishes (2×10^5^ cells per well) in DMEM containing 0–10% FBS, and incubated for different time periods. The cell numbers were counted by using trypan blue staining.

### Cell invasion assay

Cell invasion assay was performed with the modified chemotactic Boyden chamber invasion assays using 8 μm cell culture inserts. The upper chambers were coated with 100 μl diluted Matrigel (1 mg/ml). The lower chamber was filled with 600 μl 10% FBS/DMEM medium. Cell suspension (100 μL containing 3 × 10^5^ cells) was transferred to the upper chamber and incubated at 37°C for 48 h. The filter inserts were removed, fixed with methanol, and stained with Coomassie Blue for 20 minutes. Samples were subsequently washed, dried, and mounted onto slides. The invasive cells were stained blue and visualized under a microscope (Axiover Inverted Microscope, Zeiss), counted in six random fields, and the average number was recorded.

### Cell proliferation and detachment assays

4T1 cells stably transfected with miR-98, anti-miR-98, or a control vector GFP were seeded in 6-well tissue culture plates at 1×10^5^ cells per well. Cell numbers were counted on Day 2, 4 and 6. For transient transfection experiments cell number were determined on day 1, 3 and 5. For detachment experiments, 4T1 cells were seeded onto 6-well tissue culture plates at a density of 2×10^5^ cells/well in DMEM containing 5% FBS, followed by transient transfection with siRNA constructs against ALK4 and MMP11. The cells were treated with EDTA (0.01 mM) and cell detachment was analyzed by counting the detached cells.

### Colony formation in soft agarose gel

Colony formation was assessed by mixing 10^3^ cells in 0.3% low-melting agarose (Seaplaque, FMC) in DMEM supplemented with 10% FBS and plated on 0.66% agarose-coated 6-well tissue culture plates, preventing attachment of cells to the plates. The culture medium was changed twice a week with 0.5 ml DMEM containing 10% FBS. Four weeks after cell inoculation, colonies were examined and photographed under a light microscope.

### Co-culture experiments

In Ypen cell spreading experiments, miR-98-, anti-miR-98-, or GFP-transfected 4T1 cells were cultured at different cell densities in tissue culture plates overnight. Next day, Ypen cell were inoculated on top of the 4T1 cell cultures. Ypen cell spreading on top of the 4T1 cells was examined under a light and fluorescent microscope.

To test the effect of miR-98 on tube formation of Ypen cells, we mixed the miR-98, or anti-miR-98, or GFP-transfected 4T1 cells with Ypen cells. In addition, the siRNA and over-expression plasmid were also employed in the transfection experiments. Prior to mixing with each other, the transfected 4T1 cells were labeled with green fluorescent dye DiO (Invitrogen), followed by extensive wash. Ypen cells were labeled with red fluorescent dye DiI (Invitrogen), followed by extensive wash. The mixture was then cultured in Matrigel. The interaction of both types of cells and the formation of tube structures were examined under a light and fluorescent microscope.

### Tumorigenicity assays and immunohistochemistry

Five-week-old Balb/c strain mice were injected with miR-98-, anti-miR-98-, or the control vector-transfected 4T1 cells (5 × 10^5^ cells) subcutaneously. Analysis of tumorigenesis and immunohistochemistry were performed as previously described [49, 50]. A standard animal protocol was approved by the Animal Care Committee of Sunnybrook Research Institute, Ontario, Canada. Protocols established with the Animal Care Facility at Sunnybrook Research Institute dictated when mice were to be sacrificed due to humane reasons. Tumor sizes were monitored weekly thereafter. When the sizes of the tumors reached the limit allowed by the animal protocol or open tumors were seen, the mice were sacrificed and the tumors were removed.

After being sacrificed, the mice were subjected to detail examination for tumor formation. Tumors were fixed in 10% buffered formalin (Histochoice Tissue Fixative MB, Amresco), processed, and embedded in paraffin. Tumor sections were derived from the miR-98, anti-miR-98, or control tumors. Sections (4 μm thickness) were deparaffinized in 2 changes of xylene for 5 min each and rehydrated by placing the slides three times in 100% ethanol, 3 min each time, followed by staining with hematoxylin and eosin (H&E). *In situ* cell death was analyzed using the *In situ* cell death detection kit (Roche Diagnostics, Indiana polis, IN). For immunohistochemistry, endogenous peroxidase activity was blocked by incubating the sections in 3% H_2_O_2_ solution in methanol at 4°C for 20 min, followed by rinse with TBS twice, 5 min each. Antigen retrieval to unmask antigenic epitope was performed by heating the sections in sodium citrate buffer (pH 6.0) in a microwave presser cooker for 4 min. Non-specific reactions with cellular proteins were blocked with 10% normal goat serum at room temperature for 30 min. The slides were then incubated in a humidified chamber at 4°C overnight with primary antibodies (Alk4, MMP11, and CD34 prepared in TBS containing 10% normal goat serum and 1% BSA), followed by three time washes in TBS, 5 min each. The slides were then incubated with secondary antibody solution at 37°C for 45 min and with ABC (Vector labs) in the same conditions, and stained with DAB according to manufacturer's protocols. The slides were subsequently countered stained with Mayer's Hematoxylin followed by slide mounting.

### Statistical Analysis

The results (mean values ± SEM) of all the experiments were subjected to statistical analysis by *t*-test. The level of significance was set at *P* < 0.05.

## Supplementary Figures


