# Supplementary material for: MicroRNA miR-98 inhibits tumor angiogenesis and invasion by targeting activin receptor-like kinase-4 and matrix metalloproteinase-11
Source: Oncotarget. 2012 Nov 6;3(11):1370–85. doi: 10.18632/oncotarget.717 (PMC3717799; doi:10.18632/oncotarget.717)
Supplement: Supplementary file 1 [file oncotarget-03-1370-s001.pdf]

MicroRNA miR-98 inhibits tumor angiogenesis and invasion by targeting activin receptor-like kinase-4 and matrix metalloproteinase-11 - Siragam et al

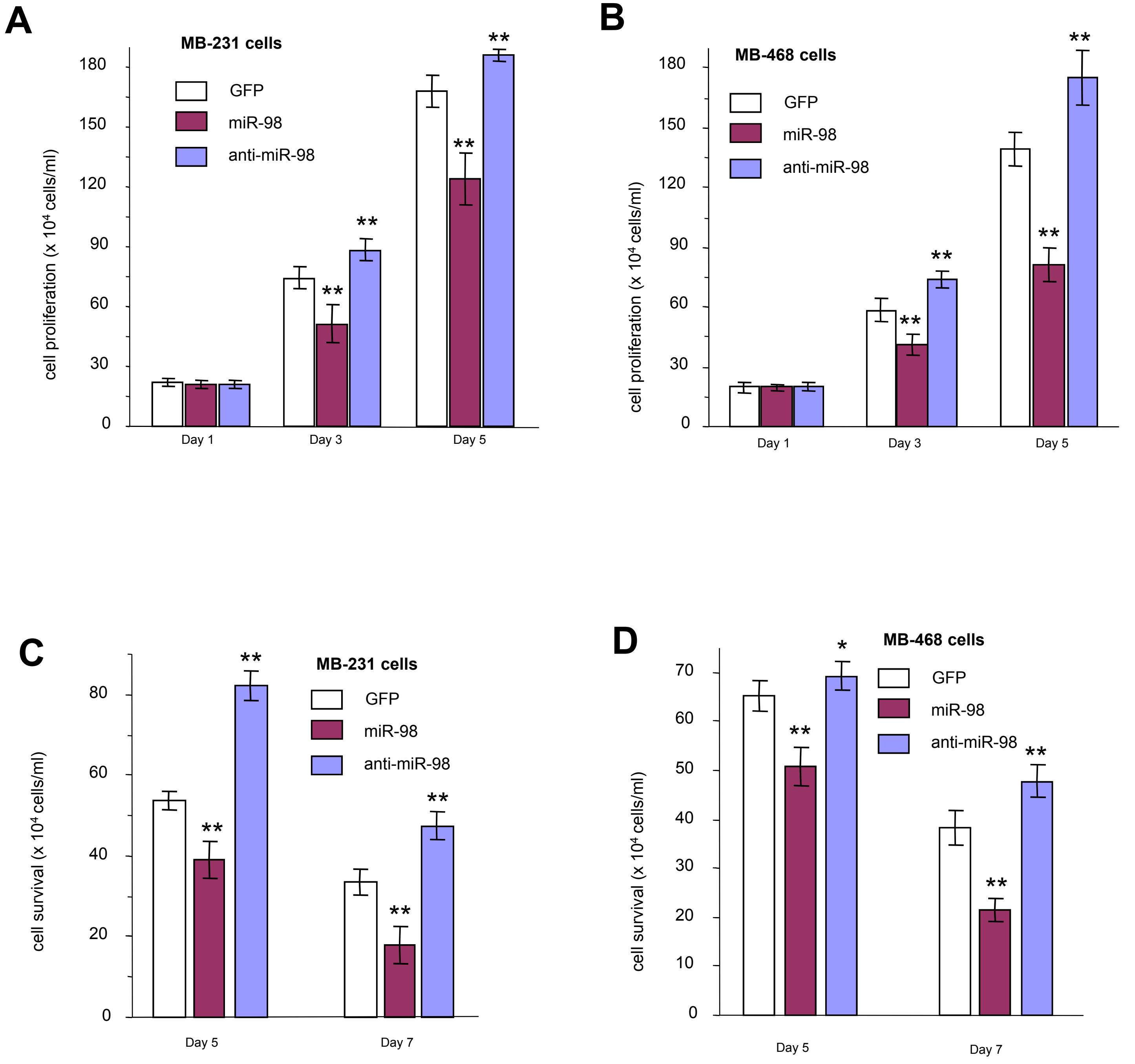

Supplementary Fig S1. Effects of miR-98 on proliferation of human breast cancer cell lines. Human breast cancer cell lines MDA-MB-231 (A) and MDA-MB-468 (B) were transiently transfected with GFP, miR-98, and anti-miR-98 for proliferation assays. Transfection with miR-98 inhibited while transfection with anti-miR-98 enhanced cell proliferation. The cells were also cultured in serum-free conditions for survival assay. Expression of miR-98 inhibited (C) while expression of anti-miR-98 enhanced (D) cell survival. Error bars indicate SEM (n=6) ***P* < 0.01, **P* < 0.05.

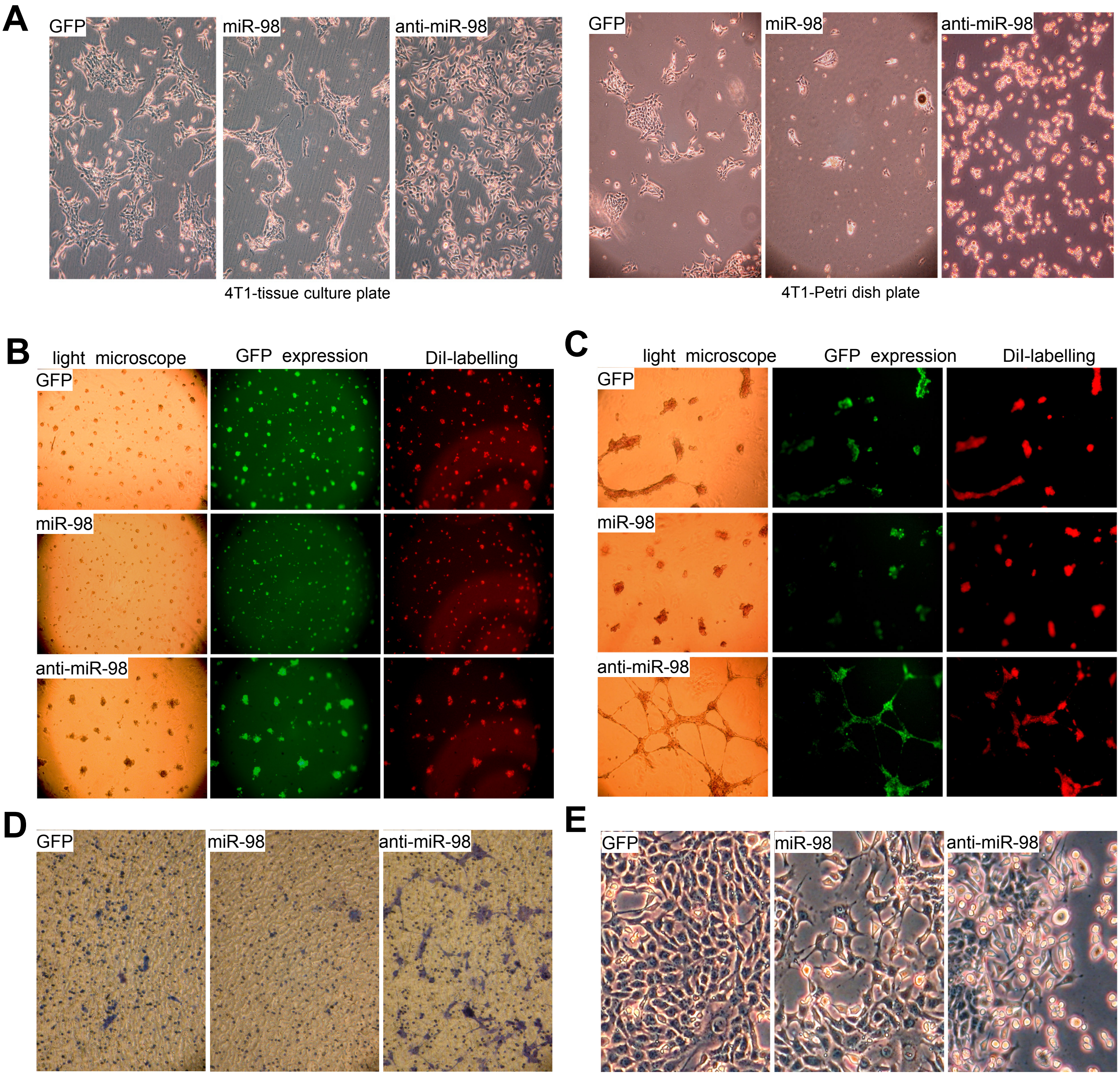

Supplementary Fig S2. Expression of miR-98 affected tube formation. (A) 4T1 cells stably transfected with miR-98, anti-miR-98, or a control vector were seeded on tissue cultures plates or Petri dishes in serum-free conditions. Cell survival was monitored by counting the viable cells. ***P*< 0.01. Error bars indicate SEM (n=4). (B) The cells were mixed with Ypen cells and inoculated in Matrigel, followed by examination of tube formation. The Ypen cells formed larger complexes and longer tubes when mixed with the anti-miR-98-expressing cells compared with the GFP- and miR-98-transfected cells. (C) Breast cancer cell line MT-1 cells were stably transfected with miR-98, anti-miR-98, or the control GFP. The cells were mixed with Ypen cells and inoculated in Matrigel, followed by tube formation assay. The Ypen cells formed smaller complexes and shorter tubes when mixed with miR-98 cells compared with GFP cells. On the other hand, the Ypen cells formed longer tubes when mixed with the anti-miR-98 cells compared with the GFP cells. (D) 4T1 cells stably transfected with miR-98, anti-miR-98, or GFP were inoculated onto Matrigel in trans-well inserts. Three days after inoculation, the cells were stained with Coomassie Blue to examine cell invasion. A typical invasion field is shown. (E) 4T1 cells stably transfected with miR-98, anti-miR-98, or a control vector were seeded on tissue cultures plates in serum-free medium. Cell morphology was monitored two days after cell inoculation under a light microscope. The anti-miR-98-transfected cells had less spreading than the control and miR-98 cells.

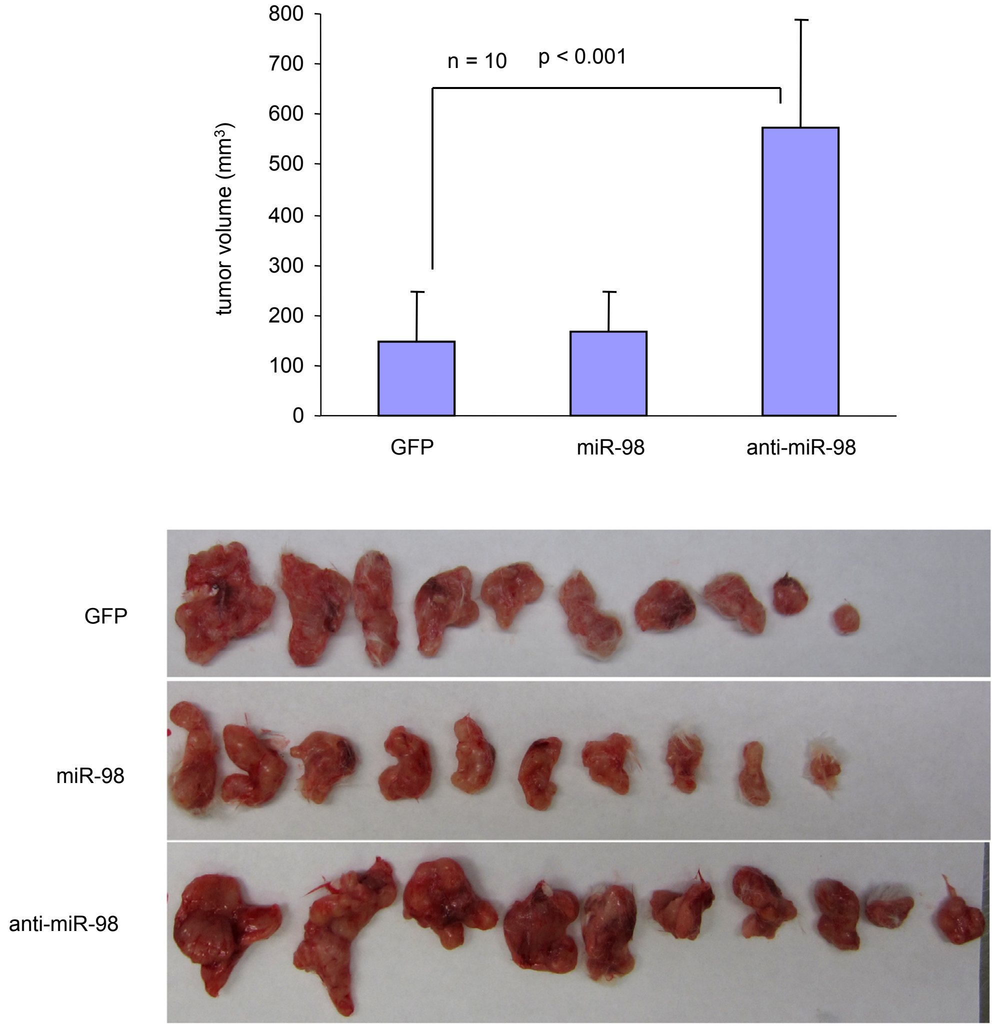

Supplementary Fig S3. Anti-miR-98 promotes tumor growth. 4T1 cells transfected with miR-98, anti-miR-98, or control GFP were injected subcutaneously into Balb/c regular mice. Tumor growth was monitored. Expression of anti-miR-98 promoted tumor growth.

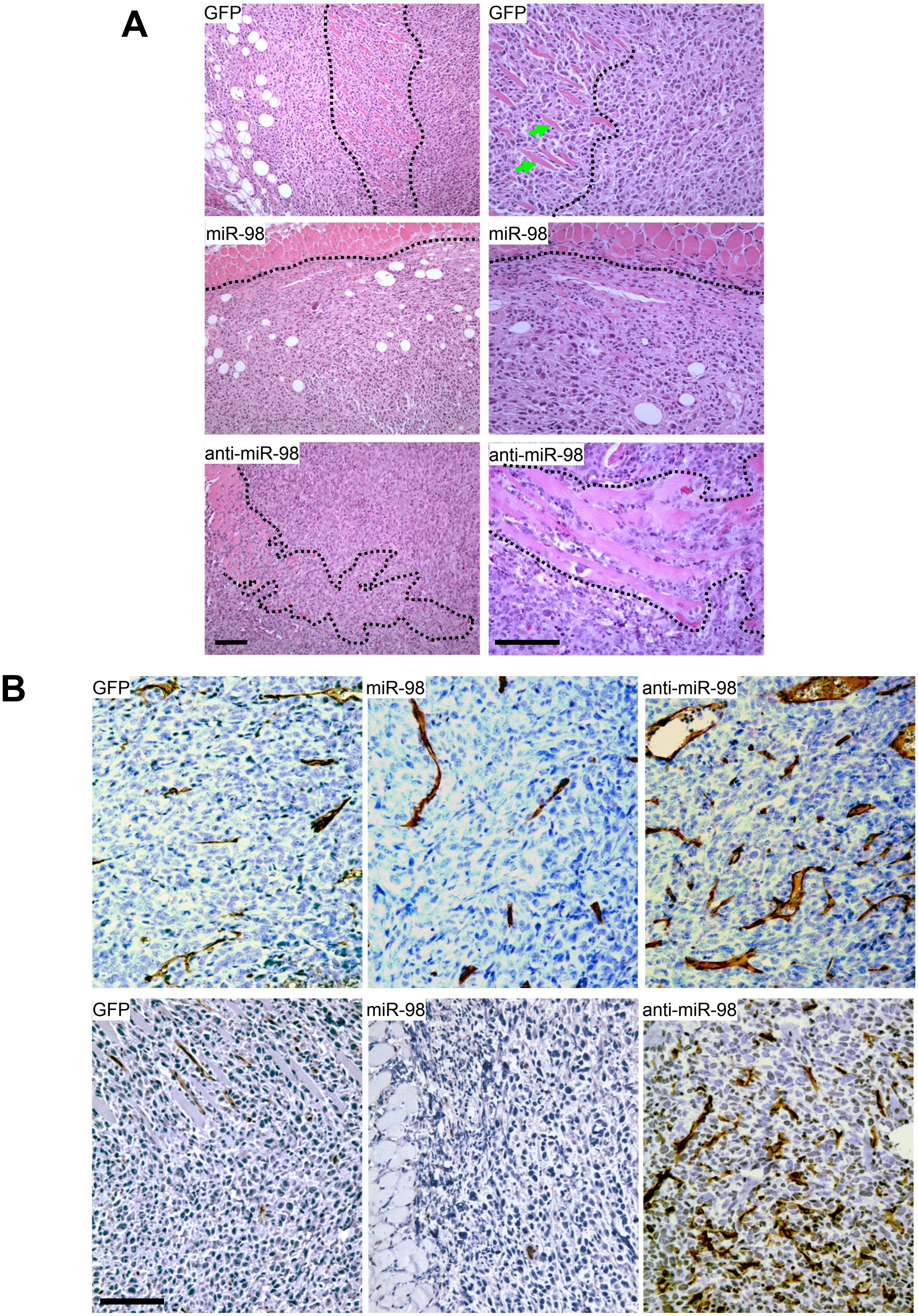

Supplementary **Fig S4. Effects of miR-98 and anti-miR-98 on Invasion and angiogenesis**. (A) The miR-98, anti-miR-98, and GFP tumors were subjected to H&E staining. Invasion of the tumor cells with stromal muscles (marked by dotted lines) occurred extensively in the anti-miR-98 cells than GFP cells. The miR-98 cells showed little invasive activity. scale bars, 100 mm. (B) Tumors formed by cells transfected with miR-98, anti-miR-98, or GFP were subjected to immunohistochemistry probed with anti-CD34 antibody to detect blood vessels (arrows). scale bar, 80 mm.

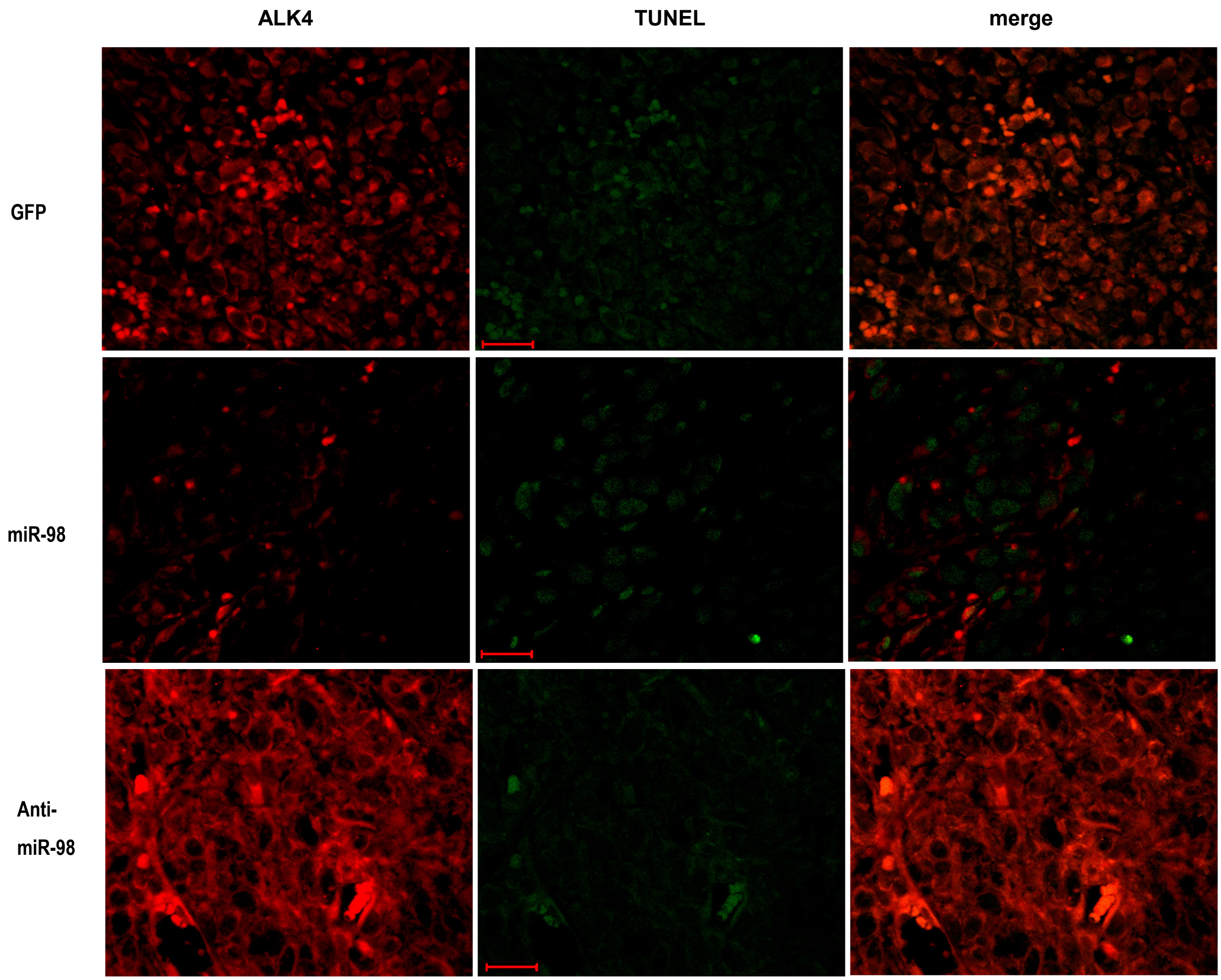

Supplementary Fig S5. ***miR-98*** **expression enhances apoptosis.** The tumor sections were immunostained for apoptotic cells (green) and for expression of ALK4 (red). The apoptotic cells were associated with cells expressing ALK4 in the miR-98 tumors. anti-miR-98 expressing cells increased expression of ALK4 but decreased apoptosis than GFP or miR-98 tumors. scale bars, 20 μM.

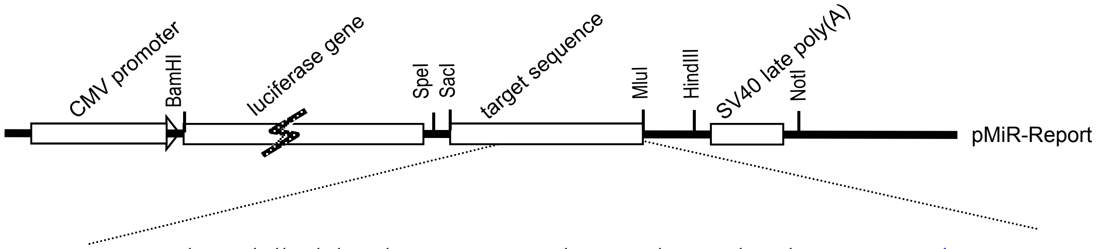

Luc-ALK4

GAGCTCtaagctgttcctctgcctacacaaagaacctgggcagtgaggatgactgcagc**caccgtgcaagc**
gtcgtggaggcctacctcttgtttctgcccggc ACGCGT

Luc-ALK4-mut

GAGCTCtaagctgttcctctgcctacacaaagaacctgggcagtgaggatgactgcagc**caccgtgcaagc**
gtcgtggaggcctaggagttgtttctgcccggc ACGCGT

Luc-MMP11

GAGCTCtgacaacactttggatgcattcaggggtactgactcctgccagggcacttagatcatgtaagagaccc
acagccatatctgtggctctgggcttcaggcatgggacagacagggcctatgtctcctcaggggagtgggttggg
gtgcagccactgtttgtaggaacgaccatgctggtcatggtcacctgccaacaattgtctcagactaggcaaagg
ctttggtgttacttaaaaataagggaggttttgggctggcaatatttcagctaccaataatccacagtcagcctggttg
cccaaggtctccatctctgtccctcaaagtagaacccccacacaaactcaggaatcacctgcaatgaggttcctg
ttgggagtggtgttggtaatgagatgcccagggtaccatgctgcccctgctaagcaactggaccagtatctttcctg
gtaagtcagctctggagagatagtgaactgatcatattctggcaggtgattcagacaagtgcttcctggaactcag
gccaaaaggtacacagccagccaaggaggcagctgcttcctcccaga**gacacggaacctcaaaggcccc**
acatacctcacagccttgccccaggcc ACGCGT

Luc-MMP11-mut

GAGCTCtgacaacactttggatgcattcaggggtactgactcctgccagggcacttagatcatgtaagagaccc
acagccatatctgtggctctgggcttcaggcatgggacagacagggcctatgtctcctcaggggagtgggttggg
gtgcagccactgtttgtaggaacgaccatgctggtcatggtcacctgccaacaattgtctcagactaggcaaagg
ctttggtgttacttaaaaataagggaggttttgggctggcaatatttcagctaccaataatccacagtcagcctggttg
cccaaggtctccatctctgtccctcaaagtagaacccccacacaaactcaggaatcacctgcaatgaggttcctg
ttgggagtggtgttggtaatgagatgcccagggtaccatgctgcccctgctaagcaactggaccagtatctttcctg
gtaagtcagctctggagagatagtgaactgatcatattctggcaggtgattcagacaagtgcttcctggaactcag
gccaaaaggtacacagccagccaaggaggcagctgcttcctcccaga**gacacggaacctcaaaggcccc**
acatacgagtcagccttgccccaggcc ACGCGT

Luc-Ctrl

actagtaatggagccacatgtatagatggcctcaatacatttacttgcctgtgtctaccaagctat
•••••••••••••••
cattttatcccatgaggaacaagtcttcgtgaaccgtattgggcacgactaccagtggattggcctcaatgaca
agatgtttgagcgtgatttc**gagctc**

Supplementary Fig S6. Generation of luciferase constructs. Fragments of ALK4 and MMP11 3'UTRs were inserted into the luciferase report vector pMir-Report producing constructs Luc-ALK4 and Luc-MMP11. The potential miR-98 target sites were labeled in blue. Mutations labeled in red were generated in the miR-98 target sites producing mutant constructs Luc-ALK4-mut and Luc-MMP11-mut.

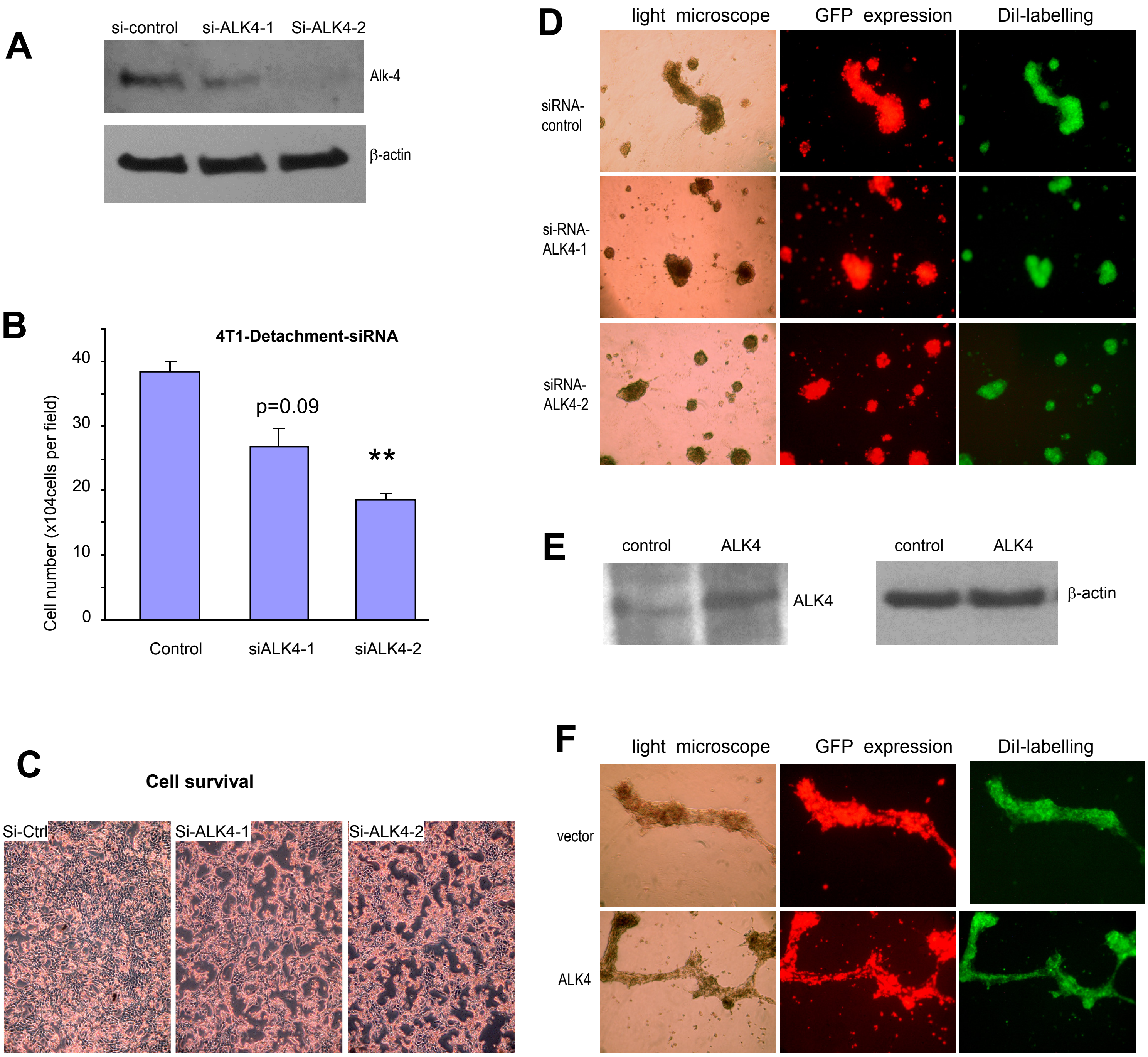

Supplementary Fig S7. Targeting ALK4 by siRNAs. (A) Cell lysates prepared from 4T1 cells transiently transfected with siRNA targeting ALK4 or a control oligo were subjected to Western blot analysis probed with anti-ALK4 antibody. (B) The 4T1 cells were transiently transfected with ALK4 siRNAs and treated with EDTA (0.01 mM). Cell detachment was examined. $^{**}P < 0.01$. (C) 4T1 cells were transiently transfected with ALK4 siRNA, or a control sequence, followed by culture in serum-free conditions for 5 days. Transfection with siRNA decreased cell survival. Typical photographs are shown and the cell number was counted for statistical analysis. (D) The 4T1 cells were transfected with ALK4 siRNAs followed by tube formation assay. Transfection with the siRNAs inhibited tube formation. (E) Cell lysates prepared from ALK4- or a control vector-transfected 4T1 cells that had been stably transfected with miR-98 or the control GFP were subjected to Western blot analysis probed with anti-ALK4 antibody. Staining for β-actin from the same membrane confirmed equal loading. (F) The miR-98 or GFP cells were transiently transfected with ALK4 or a control vector followed by tube formation assay. Expression of ALK4 promoted tube formation.

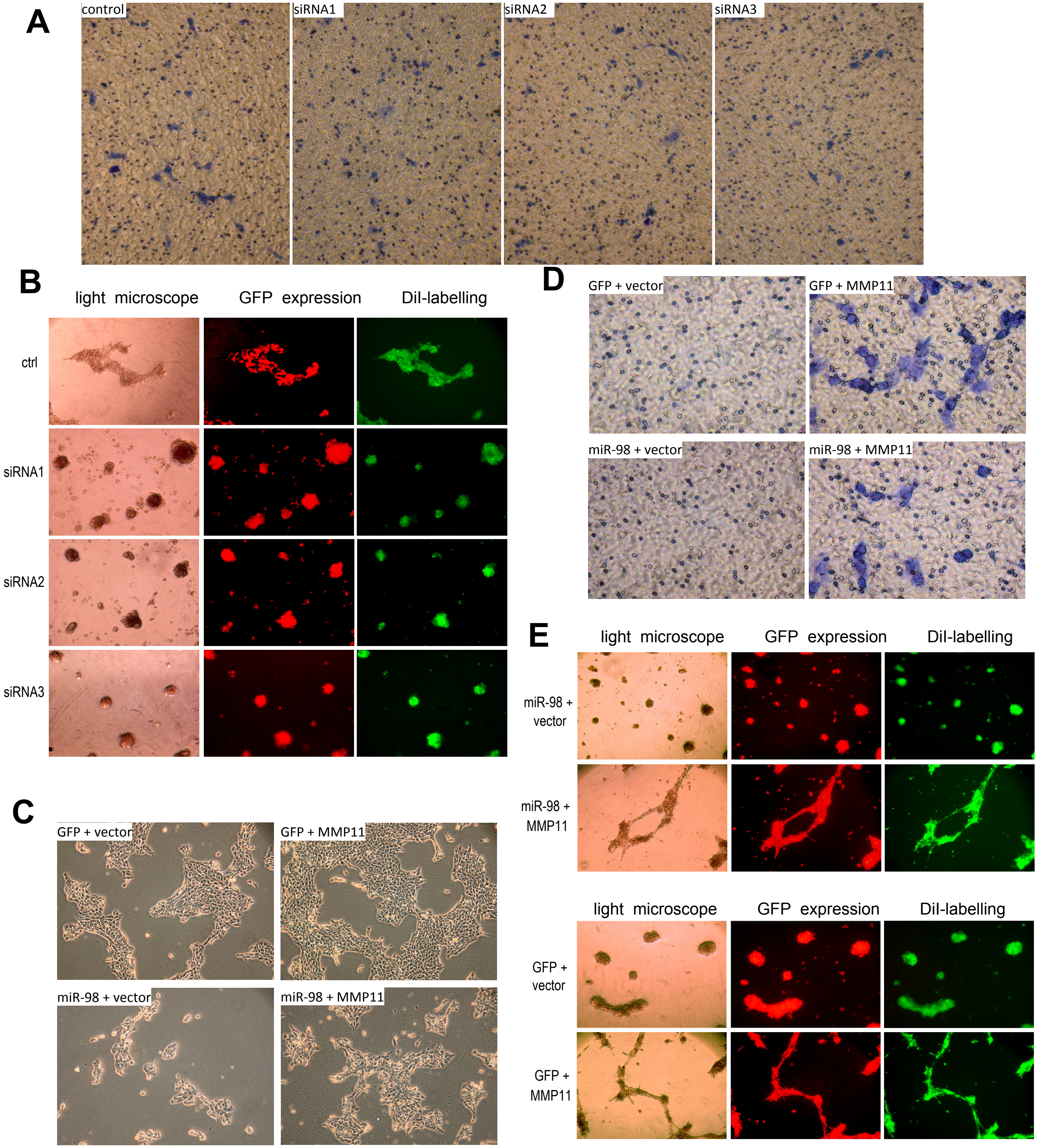

Supplementary **Fig S8. Rescue assay with MMP11 expression construct.** (A) The 4T1 cells were transfected with MMP11 siRNAs followed by cell invasion assay. Transfection with MMP11 siRNAs inhibited cell invasion. (B) The 4T1 cells were transfected with MMP11 siRNAs followed by tube formation assay. Transfection with MMP11 siRNAs inhibited tube formation. (C)The 4T1 cells stably transfected with miR-98 or GFP were transiently transfected with MMP11 or a control vector followed by culturing in serum-free conditions for 5 days. Cell survival was examined under a light microscope and photographed. (D) The miR-98 or GFP cells were transiently transfected with MMP11 or a control vector followed by cell invasion assay. Expression of MMP11 promoted cell invasion. (E) The miR-98 or GFP cells were transiently transfected with MMP11 or a control vector followed by tube formation assay. Expression of MMP11 promoted tube formation.
